# Role of metabolites in mediating the effect of lipidomes on osteoporosis: A Mendelian randomization study

**DOI:** 10.1097/MD.0000000000051393

**Published:** 2026-11-06

**Authors:** Wenyu Gao, Hao Lv, Yao Zhou, Qingsong Chu, Yan Wang, Zhimu Hu, Jiuxiang Wang, Ting Jiang

**Affiliations:** a The First Affiliated Hospital of Anhui University of Chinese Medicine, Hefei, Anhui Province, China; b Anhui University of Chinese Medicine, Hefei, Anhui Province, China; c Lujiang County Hospital of Traditional Chinese Medicine, Hefei, Anhui Province, China.

**Keywords:** mediation analysis, Mendelian randomization, osteoporosis, plasma lipidomes, plasma metabolites

## Abstract

We investigated the causal relationships between the plasma lipidomes, osteoporosis (OP), and potential metabolite mediators using Mendelian randomization (MR). Using summary-level data from a genome-wide association study, bidirectional MR analyses to explore the causal relationships between the plasma lipidomes and OP, and two-step MR to discover potential mediating metabolites. Sensitivity analyses were performed to validate the robustness of the results further. After primary analysis and a series of sensitivity analyses, 7 inflammation-related proteins were identified that have a causal relationship with OP. Ceramide (d40:1) levels (odds ratio [OR] = 0.923; 95% confidence intervals (CI): 0.854–0.999; *P* = .046 < .05), phosphatidylcholine (16:0_18:2) levels (OR = 0.932; 95% CI: 0.883–0.985; *P* = .012 < .05), phosphatidylc holine (16:1_18:2) levels (OR = 0.939; 95% CI: 0.882–0.999; *P* = .047 < .05), triacylglycerol (48:3) levels (OR = 0.920; 95% CI: 0.854–0.971; *P* = .027 < .05), and triacylglycerol (51:4) levels (OR = 0.914; 95% CI: 0.840–0.995; *P* = .038 < .05) were negatively associated with OP, whereas phosphatidylcholine (O-17:0_15:0) levels (OR = 1.121; 95% CI: 1.019–1.233; *P* = .019 < .05), and triacylglycerol (48:0) levels (OR = 1.130; 95% CI: 1.042–1.226; *P* = .003 < .05) were positively associated with OP. Subsequently, we conducted a two-step MR to discover potential mediating metabolites. This study found that 14 plasma metabolites may play a mediating role in the relationship between plasma lipidomes and OP, such as 5alpha-androstan-3alpha,17beta-diol monosulfate (1) levels, N6-methyllysine levels, 5-oxoproline levels, 2-hydroxyhippurate levels, glycosyl ceramide (d18:1/20:0, d16:1/22:0) levels, spermidine to 5-methylthioadenosine ratio, serine levels, phosphate to serine ratio, proline to trans-4-hydroxyproline ratio, 1-palmitoyl-GPC (16:0) levels, 7-methylxanthine levels, arachidonoylcholine levels, and spermidine to choline ratio. In conclusion, our study identified a causal relationship between plasma lipidomes and OP, with a proportion of the effect mediated by plasma metabolites.

## 1. Introduction

Osteoporosis (OP) is a condition that universally affects people of all ethnic backgrounds, especially many older women and men. It is characterized by decreased bone mass, deterioration of microstructure, and susceptibility to fragility fractures.^[1]^ It is the most common metabolic bone disease in the world. It is usually an asymptomatic subclinical state until a fracture occurs. Osteoporosis requires long-term or even lifelong management, especially in individuals at high risk of fracture.^[2]^ Comprehending the causal links between OP and potential risk factors like plasma lipidomes and metabolites is essential for formulating efficient prevention and treatment approaches.

Plasma lipidomes are complex mixtures of endogenous lipids circulating in the blood, which reflect systemic metabolic states and have been implicated in various diseases, including bone disorders. Their composition and levels can influence cellular signaling, membrane properties, and energy metabolism, thereby potentially modulating bone remodeling processes. Several studies have investigated the relationship between circulating lipids and bone mineral density, but the causal nature and the underlying mechanisms remain unclear.^[3,4]^ In particular, whether certain lipid species directly affect OP risk or act indirectly through intermediate metabolites is yet to be systematically evaluated.

Plasma metabolites, small molecules produced during metabolic processes, play a crucial role in reflecting the physiological and pathological states of the body, including the profiling of pathways in noncommunicable disease multimorbidity as highlighted by Pietzner et al.^[5]^ These metabolites are interconnected with lipidomes, as Kuznetsova and Vodovozova demonstrated the differential binding of plasma proteins by lipidomes loaded with lipophilic prodrugs, affecting drug delivery efficiency and efficacy.^[6]^ Furthermore, plasma metabolites have been linked to osteoporosis (OP) risk, with research by Di et al providing insights into the relationship between metal mixtures and OP through plasma metabolite profiling,^[7]^ and Mangano et al discovering sex-specific inverse relationships between diet-derived fruit and vegetable metabolites and OP status.^[8]^ Collectively, these studies underscore the complex interplay between plasma metabolites, lipidomes, and OP, offering new perspectives on the mechanisms underlying disease progression and potential therapeutic targets, as well as the importance of plasma lipid metabolites in identifying individuals at risk of obesity-induced metabolic complications.^[9]^

Thus, we hypothesized that there might be causal associations between plasma lipidomes, blood metabolites, and OP, and that metabolites might mediate part of the lipid-OP relationship. However, previous studies have largely focused on single omics layers without integrating them into a causal mediation framework. Therefore, our study aims to clarify these associations and identify potential metabolomic mediators that could serve as early diagnostic biomarkers or therapeutic targets. By employing bidirectional and two-step MR, we seek to disentangle the direct and indirect pathways linking lipidomes to OP.

The genome-wide association studies (GWAS) have greatly aided in the identification of single nucleotide polymorphisms (SNPs) linked to common complex diseases.^[10]^ Mendelian randomization (MR) analysis uses genetic variation as an instrumental variable to investigate the relationship between exposure factors and disease outcomes.^[11]^ MR, compared to traditional observational studies, enables more precise and reliable causal inference.^[12]^ In this study, we performed a bidirectional MR study and mediation analyses using summary statistics of the plasma lipidomes, blood metabolites, and OP to dissect the associations between them.

## 2. Materials and methods

### 2.1. Study design

Figure 1 provides a schematic overview of the study design. Initially, we designated the plasma lipidomes as the exposure to ascertain their potential roles in either inhibiting or fostering the onset of OP. OP was then used as the exposure and plasma lipidomes as the outcome to investigate changes in metabolites after the occurrence of the disease. Then plasma metabolites are used as mediators to further evaluate this relationship.

**Figure 1.**
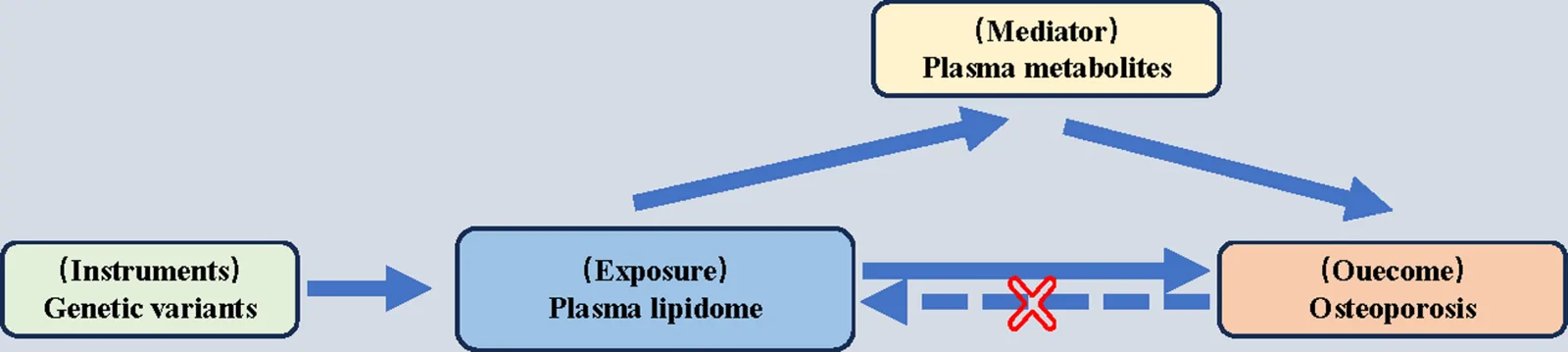
Assumptions and design of the bidirectional and mediation MR analyses. Firstly, a two-sample bidirectional MR was performed to investigate the causal relationships between plasma lipidomes (exposure) and OP (outcome). Secondly, plasma metabolites (mediators) were selected for subsequent mediation analyses. Finally, a two-step MR analysis was conducted to detect potential mediating metabolites (Step 1, the effect of plasma lipidomes on metabolites; Step 2, the effect of metabolites on OP). MR = Mendelian randomization, OP = osteoporosis.

### 2.2. Data sources and study samples

In this study, we utilized publicly available GWAS summary data for inflammation-related proteins, plasma metabolites, and OP-related traits. Data on OP were drawn from the GWAS summary data sources on the FennGenn consortium, which is available at https://www.finngen.fi/en (OP including 3203 cases and 209,575 controls). Data for the GWAS of 179 plasma lipidomes and 1400 plasma metabolites were obtained from published studies.^[13,14]^ The summary-level data utilized in this study can be downloaded and has been obtained with ethical approval from the respective institutions overseeing each GWAS involved. The source of GWAS summary data can be found in Supplementary Table 1, Supplemental Digital Content 1. Importantly, the exposure and outcome datasets are derived from independent GWAS studies, ensuring that there is no sample overlap, which is a core assumption for two-sample MR.

### 2.3. Instrumental variables selection

IVs were selected based on the following criteria: strong genome-wide association with the exposure metabolites, independent of potential confounders, and not associated with other intermediate variables in the causal pathway. We selected IVs using a *P* value threshold of 5 × 10^−8^.

To ensure independence, we performed linkage disequilibrium clumping using a threshold of *r*^2^ < 0.001 within a 10,000 kb window. We assessed instrument strength by calculating the *F*-statistic for each SNP; all had *F*-statistics > 10, indicating no weak instrument bias. Palindromic SNPs with ambiguous allele frequencies were handled using the harmonise_data function in the TwoSampleMR package, and any incompatible SNPs were excluded.

### 2.4. Mendelian randomization analysis

The primary strategy utilized in this study to evaluate the initial connection between exposure and outcome was the inverse variance weighted (IVW) method, which is the best unbiased estimate in the absence of pleiotropy.^[15]^ IVW is a method widely used in MR Research that estimates causal effects by weighting the Wald ratios of each SNP.^[16]^ In addition, we also estimated the effects using 4 MR methods (MR-Egger, weighted median, simple mode, and weighted mode). We conducted a bidirectional MR analysis involving 2 independent samples to assess the reciprocal causal relationship between plasma lipidomes and OP. Moreover, we employed a two-step MR design to conduct a mediation analysis, aiming to investigate whether plasma metabolites act as mediators in the causal pathway from plasma lipidomes to OP.

For the two-step mediation analysis, we first estimated the effect of each lipidome on each metabolite (Step 1: β1), and then the effect of each metabolite on OP (Step 2: β2). The indirect effect (β_indirect) was calculated as β1 × β2, and the proportion mediated was computed as (β_indirect/ β_total) × 100%, where β_total is the total effect of lipidome on OP. Negative proportions were interpreted as suppression effects rather than true mediation.

### 2.5. Sensitivity analysis

The heterogeneity between SNPs was evaluated by Cochran *Q* test. Horizontal pleiotropy was assessed through the application of the MR-Egger intercept method and the MR-Pleiotropy RESidual Sum and Outlier (PRESSO) method. In cases where outliers were identified, they were excluded from the analysis, followed by a reevaluation of the MR causal estimates. To further validate the impact of each SNP on the overall causal estimates, a leave-one-out analysis was performed.

### 2.6. Statistical analysis

Analyses were executed using R statistical software (version 4.2.3, http://www.r-project.org). MR analyses were conducted with the R-based tools “TwoSampleMR and “MR-PRESSO.”^[17,18]^ Given the multiple testing issue, we applied false discovery rate correction to the primary analyses. For the exploratory mediation analyses, we report nominal *P*-values and interpret results as hypothesis-generating.

## 3. Results

### 3.1. Subsection 1: primary bidirectional MR analysis – causal association of plasma lipidomes with OP

As shown in Table 1 and Figure 2, according to the results of IVW, we found that ceramide (d40:1) levels (odds ratio [OR] = 0.923; 95% confidence intervals (CI): 0.854–0.999; *P* = .046 < .05; *q* = 0.051), phosphatidylcholine (16:0_18:2) levels (OR = 0.932; 95% CI: 0.883–0.985; *P* = .012 < .05; *q* = 0.021), phosphatidylcholine (16:1_18:2) levels (OR = 0.939; 95% CI: 0.882–0.999; *P* = .047 < .05; *q* = 0.052), triacylglycerol (48:3) levels (OR = 0.920; 95% CI: 0.854–0.971; *P* = .027 < .05; *q* = 0.038), and triacylglycerol (51:4) levels (OR = 0.914; 95% CI: 0.840–0.995; *P* = .038 < .05; *q* = 0.049) were negatively associated with OP, whereas phosphatidylcholine (O-17:0_15:0) levels (OR = 1.121; 95% CI: 1.019–1.233; *P* = .019 < .05; *q* = 0.032), and triacylglycerol (48:0) levels (OR = 1.130; 95% CI: 1.042–1.226; *P* = .003 < .05; *q* = 0.009) were positively associated with OP.

**Table 1:** MR estimates of assessing the causal effect of plasma lipidomes on OP.

Outcome	Exposure	Method	Number of SNPs	Beta	SE	*P* value	OR (95% CI)
OP	Ceramide (d40:1) levels	IVW	28	−0.080	0.040	.046	0.923 (0.854–0.999)
OP	Phosphatidylcholine (16:0_18:2) levels	IVW	36	−0.070	0.028	.012	0.932 (0.883–0.985)
OP	Phosphatidylcholine (16:1_18:2) levels	IVW	25	−0.063	0.032	.047	0.939 (0.882–0.999)
OP	Phosphatidylcholine (O-17:0_15:0) levels	IVW	19	0.114	0.049	.019	1.121 (1.019–1.233)
OP	Triacylglycerol (48:0) levels	IVW	19	0.122	0.041	.003	1.130 (1.042–1.226)
OP	Triacylglycerol (48:3) levels	IVW	27	−0.084	0.038	.027	0.920 (0.854–0.991)
OP	Triacylglycerol (51:4) levels	IVW	20	−0.090	0.043	.038	0.914 (0.840–0.995)

CI = confidence interval, IVW = inverse variance weighted, OP = osteoporosis, OR = odds ratio, SNP = single nucleotide polymorphism.

**Figure 2.**
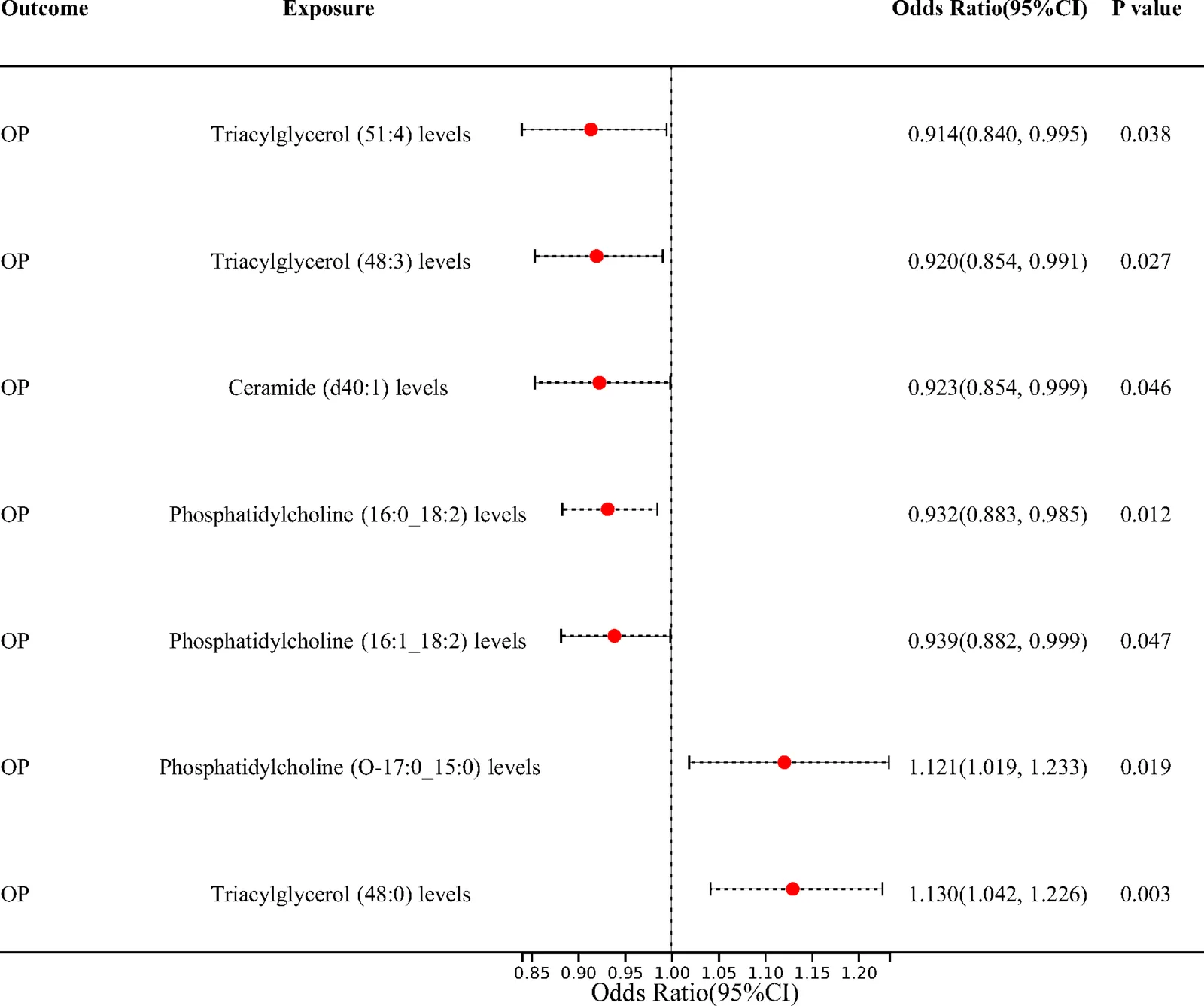
The forest plot of MR analyses the causal effect of plasma lipidomes on OP. Data are presented as odds ratios (OR) with 95% confidence intervals (CI) for the association between each plasma lipidome and osteoporosis risk. The inverse variance weighted (IVW) method was used as the primary analysis. Error bars represent 95% CIs. Abbreviations: Cer(d40:1), ceramide (d40:1) levels; PC(16:0_18:2), phosphatidylcholine (16:0_18:2) levels; PC(16:1_18:2), phosphatidylcholine (16:1_18:2) levels; PC(O-17:0_15:0), phosphatidylcholine (O-17:0_15:0) levels; TG(48:0), triacylglycerol (48:0) levels; TG(48:3), triacylglycerol (48:3) levels; TG(51:4), triacylglycerol (51:4) levels. MR = Mendelian randomization, OP = osteoporosis.

Cautionary note: ceramide (d40:1, *P* = .046), phosphatidylcholine (16:1_18:2, *P* = .047), and triacylglycerol (51:4, *P* = .038) are borderline nominally significant associations that fail to meet Bonferroni-adjusted threshold, these findings carry limited causal evidence strength and require independent cohort replication. Only triacylglycerol (48:0) reached robust statistical significance after multiple testing correction.

Cochran *Q* test showed SNPs of ceramide (d40:1) levels were heterogeneous (Table 2 and Fig. 3). So the random-effects IVW approach was utilized to analyze the causal effect of these plasma metabolites on OP. The MR-Egger intercept method and the MR-PRESSO results indicated no horizontal pleiotropy across SNPs in the causal estimates (Table 3). As shown in Table 4, the IVW results showed OP did not causal effect on plasma lipidomes.

**Table 2:** Heterogeneity analyses of the causal association between plasma lipidomes and OP.

Outcome	Exposure	Heterogeneity test			
		MR-Egger		IVW	
		*Q*	*P* value	*Q*	*P* value
OP	Ceramide (d40:1) levels	26	.045	27	.052
OP	Phosphatidylcholine (16:0_18:2) levels	34	.308	35	.343
OP	Phosphatidylcholine (16:1_18:2) levels	23	.292	24	.260
OP	Phosphatidylcholine (O-17:0_15:0) levels	17	.205	18	.204
OP	Triacylglycerol (48:0) levels	17	.824	18	.833
OP	Triacylglycerol (48:3) levels	25	.358	26	.400
OP	Triacylglycerol (51:4) levels	18	.224	19	.093

IVW = inverse variance weighted, OP = osteoporosis.

**Table 3:** Pleiotropy analyses of the causal association between plasma lipidomes and OP.

Outcome	Exposure	Pleiotropy test						
		MR-Egger pleiotropy test			MR-PRESSO distorted outlier test			
		Egger intercept	SE	*P* value	MR-PRESSO global test *P* value	Outlier-corrected *P* value	Outliers indices	Distortion test *P* value
OP	Ceramide (d40:1) levels	−0.007	0.012	.549	.078	NA	NA	NA
OP	Phosphatidylcholine (16:0_18:2) levels	−0.004	0.009	.681	.371	NA	NA	NA
OP	Phosphatidylcholine (16:1_18:2) levels	−0.011	0.009	.218	.299	NA	NA	NA
OP	Phosphatidylcholine (O-17:0_15:0) levels	0.014	0.014	.349	.215	NA	NA	NA
OP	Triacylglycerol (48:0) levels	−0.010	0.012	.424	.849	NA	NA	NA
OP	Triacylglycerol (48:3) levels	0.006	0.014	.664	.424	NA	NA	NA
OP	Triacylglycerol (51:4) levels	−0.029	0.014	.052	.095	NA	NA	NA

MR = Mendelian randomization, OP = osteoporosis, PRESSO = Pleiotropy RESidual Sum and Outlier, SE = standard error.

**Table 4:** MR estimates of assessing the causal effect of OP on plasma lipidomes.

Outcome	Exposure	Method	Number of SNPs	Beta	SE	*P* value	OR (95% CI)
Ceramide (d40:1) levels	OP	IVW	33	0.002	0.036	.963	1.002 (0.934–1.075)
Phosphatidylcholine (16:0_18:2) levels	OP	IVW	33	0.007	0.036	.852	1.007 (0.939–1.08)
Phosphatidylcholine (16:1_18:2) levels	OP	IVW	33	−0.034	0.037	.360	0.967 (0.899–1.04)
Phosphatidylcholine (O-17:0_15:0) levels	OP	IVW	33	−0.009	0.039	.807	0.991 (0.918–1.068)
Triacylglycerol (48:0) levels	OP	IVW	33	0.009	0.050	.852	1.009 (0.915–1.114)
Triacylglycerol (48:3) levels	OP	IVW	33	−0.050	0.043	.240	0.951 (0.874–1.034)
Triacylglycerol (51:4) levels	OP	IVW	33	−0.042	0.051	.408	0.959 (0.868–1.059)

CI = confidence interval, IVW = inverse variance weighted, MR = Mendelian randomization, OP = osteoporosis, OR = odds ratio, SE = standard error.

**Figure 3.**
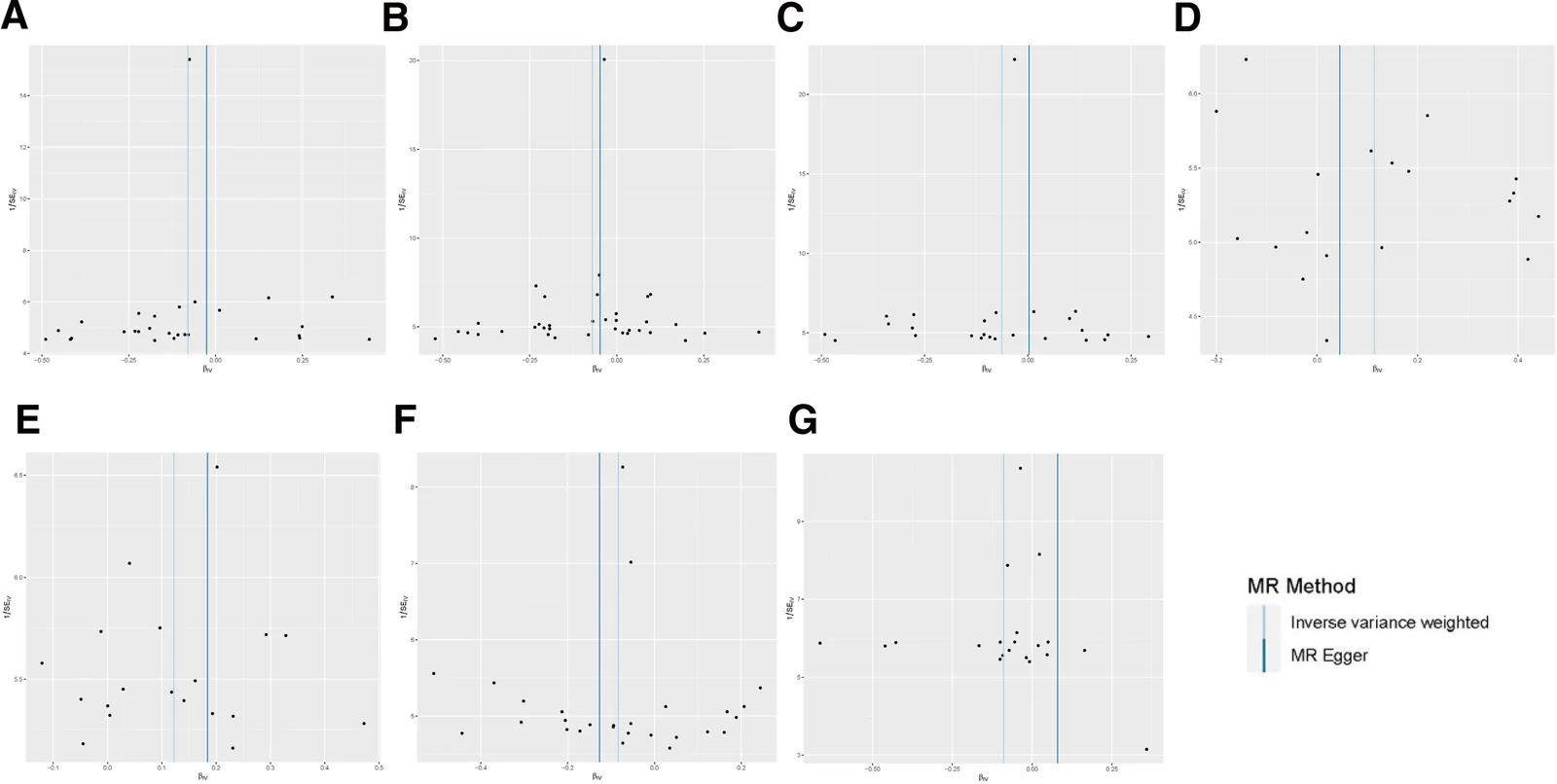
Funnel plots were applied to detect whether the observed association was along with obvious heterogeneity. (A) Ceramide (d40:1) levels on OP. (B) Phosphatidylcholine (16:0_18:2) levels on OP. (C) Phosphatidylcholine (16:1_18:2) levels on OP. (D) Phosphatidylcholine (O-17:0_15:0) levels on OP. (E) Triacylglycerol (48:0) levels on OP. (F) Triacylglycerol (48:3) levels on OP. (G) Triacylglycerol (51:4) levels on OP. Each dot represents a single SNP; the dashed vertical line indicates the overall causal estimate using IVW method. Symmetrical distribution around the estimate suggests absence of heterogeneity. IVW = inverse variance weighted, OP = osteoporosis, SNP = single nucleotide polymorphism.

### 3.2. Subsection 2: secondary screening – lipidome-metabolite and metabolite-OP causal pairwise associations

A total of 582 plasma metabolites were causally associated with these 7 plasma lipidomes after false discovery rate correction (Supplementary Table 2, Supplemental Digital Content 2). Based on the screening results, 29 plasma metabolites showed nominal causal associations with OP via IVW analysis (Supplementary Table 3, Supplemental Digital Content 3), laying the foundation for subsequent mediation testing.

### 3.3. Subsection 3: exploratory two-step MR mediation analysis of mediating plasma metabolites

We then performed mediation analyses. The following results are exploratory and should be viewed as hypothesis-generating; they have not been corrected for multiple testing. We analyzed plasma metabolites as mediators of the pathway from plasma lipidomes to OP. As shown in Table 5 and Figures 4 and 5, our study showed that glycosyl ceramide (d18:1/20:0, d16:1/22:0) levels and phosphate to serine ratio could account for the negative association between ceramide (d40:1) levels and OP. 7-Methylxanthine levels, serine levels, and X-23739 levels could decrease the increased negative association between ceramide (d40:1) levels and OP. Glycosyl ceramide (d18:1/20:0, d16:1/22:0) levels and 5-oxoproline levels could account for the negative relationship between phosphatidylcholine (16:0_18:2) levels and OP. arachidonoylcholine levels and arachidonoylcholine levels could decrease the negative relationship between phosphatidylcholine (16:0_18:2) and OP. Proline to trans-4-hydroxyproline ratio could decrease the increased risk of phosphatidylcholine (O-17:0_15:0) associated with OP. 2-hydroxyhippurate levels could decrease the increased risk of triacylglycerol (48:0) levels associated with OP. 1-palmitoyl-GPC (16:0) levels and 5alpha-androstan-3alpha,17beta-diol monosulfate (1) levels could account for the negative relationship between triacylglycerol (48:3) levels and OP. N6-methyllysine levels could account for the negative relationship between triacylglycerol (51:4) levels and OP. Spermidine to 5-methylthioadenosine (MTA) ratio and triacylglycerol (51:4) levels could decrease the negative relationship between triacylglycerol (51:4) levels and OP.

**Table 5:** The two-step MR analyses of the causal effects between plasma lipidomes, plasma metabolites, and OP.

Outcome	Exposure	Mediators	beta all ± SE	beta1 ± SE	beta2 ± SE	beta indirect ± SE	Proportion mediated (95% CI)
OP	Ceramide (d40:1) levels	Trigonelline levels	−0.08 ± 0.04	−0.066 ± 0.031	−0.085 ± 0.037	0.006 ± 0.004	NA
OP	Ceramide (d40:1) levels	7-Methylxanthine levels	−0.08 ± 0.04	−0.079 ± 0.032	0.158 ± 0.051	−0.012 ± 0.008	0.156 (0.262–0.05)
OP	Ceramide (d40:1) levels	Glycosyl ceramide (d18:1/20:0, d16:1/22:0) levels	−0.08 ± 0.04	0.19 ± 0.056	−0.084 ± 0.033	−0.016 ± 0.001	0.201 (0.34–0.063)
OP	Ceramide (d40:1) levels	Adenosine 3’,5’-cyclic monophosphate (camp) levels	−0.08 ± 0.04	−0.078 ± 0.038	0.066 ± 0.032	0.007 ± 0.005	NA
OP	Ceramide (d40:1) levels	Serine levels	−0.08 ± 0.04	−0.114 ± 0.038	0.066 ± 0.032	−0.008 ± 0.005	0.094 (0.154–0.034)
OP	Ceramide (d40:1) levels	X-23739 levels	−0.08 ± 0.04	−0.078 ± 0.038	0.123 ± 0.048	−0.01 ± 0.007	0.12 (0.203–0.037)
OP	Ceramide (d40:1) levels	Phosphate to serine ratio	−0.08 ± 0.04	0.096 ± 0.036	−0.086 ± 0.04	−0.008 ± 0.005	0.103 (0.164–0.042)
OP	Phosphatidylcholine (16:0_18:2) levels	3-Hydroxydodecanedioate levels	−0.07 ± 0.028	0.068 ± 0.028	0.084 ± 0.041	0.006 ± 0.004	NA
OP	Phosphatidylcholine (16:0_18:2) levels	1,2-Dilinoleoyl-GPE (18:2/18:2) levels	−0.07 ± 0.028	0.257 ± 0.057	0.083 ± 0.038	0.021 ± 0.015	NA
OP	Phosphatidylcholine (16:0_18:2) levels	Arachidonoylcholine levels	−0.07 ± 0.028	−0.134 ± 0.052	0.123 ± 0.044	−0.016 ± 0.009	0.235 (0.36–0.109)
OP	Phosphatidylcholine (16:0_18:2) levels	Glycosyl ceramide (d18:1/20:0, d16:1/22:0) levels	−0.07 ± 0.028	0.067 ± 0.025	−0.084 ± 0.033	−0.006 ± 0.003	0.081 (0.127–0.035)
OP	Phosphatidylcholine (16:0_18:2) levels	5-Oxoproline levels	−0.07 ± 0.028	0.05 ± 0.024	−0.067 ± 0.034	−0.003 ± 0.003	0.048 (0.085–0.011)
OP	Phosphatidylcholine (16:0_18:2) levels	Adenosine 3’,5’-cyclic monophosphate (camp) levels	−0.07 ± 0.028	0.076 ± 0.026	0.066 ± 0.032	0.005 ± 0.003	NA
OP	Phosphatidylcholine (16:0_18:2) levels	Serine levels	−0.07 ± 0.028	0.056 ± 0.024	0.066 ± 0.032	0.004 ± 0.003	NA
OP	Phosphatidylcholine (16:0_18:2) levels	Adenosine 5’-monophosphate (AMP) to phosphate ratio	−0.07 ± 0.028	0.05 ± 0.024	0.115 ± 0.054	0.006 ± 0.006	NA
OP	Phosphatidylcholine (16:0_18:2) levels	Phosphate to threonine ratio	−0.07 ± 0.028	−0.063 ± 0.023	−0.088 ± 0.038	0.006 ± 0.004	NA
OP	Phosphatidylcholine (16:0_18:2) levels	Phosphate to serine ratio	−0.07 ± 0.028	−0.087 ± 0.024	−0.086 ± 0.04	0.007 ± 0.004	NA
OP	Phosphatidylcholine (16:1_18:2) levels	Indolebutyrate levels	−0.063 ± 0.032	0.103 ± 0.047	0.076 ± 0.038	0.008 ± 0.006	NA
OP	Phosphatidylcholine (16:1_18:2) levels	S-methylcysteine sulfoxide levels	−0.063 ± 0.032	−0.056 ± 0.028	−0.117 ± 0.046	0.007 ± 0.006	NA
OP	Phosphatidylcholine (16:1_18:2) levels	1,2-Dilinoleoyl-GPE (18:2/18:2) levels	−0.063 ± 0.032	0.355 ± 0.073	0.083 ± 0.038	0.03 ± 0.026	NA
OP	Phosphatidylcholine (16:1_18:2) levels	Arachidonoylcholine levels	−0.063 ± 0.032	−0.225 ± 0.06	0.123 ± 0.044	−0.028 ± 0.015	0.439 (0.669–0.209)
OP	Phosphatidylcholine (16:1_18:2) levels	8-Methoxykynurenate levels	−0.063 ± 0.032	−0.071 ± 0.034	−0.119 ± 0.04	0.008 ± 0.005	NA
OP	Phosphatidylcholine (16:1_18:2) levels	X-12216 levels	−0.063 ± 0.032	−0.076 ± 0.028	−0.091 ± 0.044	0.007 ± 0.005	NA
OP	Phosphatidylcholine (O-17:0_15:0) levels	Myristoleate (14:1n5) levels	0.114 ± 0.049	0.068 ± 0.034	−0.126 ± 0.053	−0.008 ± 0.007	NA
OP	Phosphatidylcholine (O-17:0_15:0) levels	2-Hydroxyhippurate levels	0.114 ± 0.049	0.107 ± 0.038	−0.116 ± 0.051	−0.012 ± 0.007	NA
OP	Phosphatidylcholine (O-17:0_15:0) levels	Proline to trans-4-hydroxyproline ratio	0.114 ± 0.049	−0.148 ± 0.047	−0.092 ± 0.046	0.014 ± 0.008	0.12 (0.049–0.191)
OP	Triacylglycerol (48:0) levels	5alpha-androstan-3alpha,17beta-diol monosulfate (1) levels	0.122 ± 0.041	0.12 ± 0.041	−0.048 ± 0.019	−0.006 ± 0.005	NA
OP	Triacylglycerol (48:0) levels	2-Hydroxyhippurate levels	0.122 ± 0.041	−0.077 ± 0.036	−0.116 ± 0.051	0.009 ± 0.007	0.073 (0.019–0.126)
OP	Triacylglycerol (48:0) levels	N6,N6-dimethyllysine levels	0.122 ± 0.041	0.077 ± 0.036	−0.054 ± 0.025	−0.004 ± 0.003	NA
OP	Triacylglycerol (48:3) levels	1-Palmitoyl-GPC (16:0) levels	−0.084 ± 0.038	0.111 ± 0.055	−0.097 ± 0.043	−0.011 ± 0.007	0.129 (0.217–0.04)
OP	Triacylglycerol (48:3) levels	5alpha-androstan-3alpha,17beta-diol monosulfate (1) levels	−0.084 ± 0.038	0.074 ± 0.036	−0.048 ± 0.019	−0.004 ± 0.003	0.043 (0.076–0.01)
OP	Triacylglycerol (48:3) levels	Nicotinamide levels	−0.084 ± 0.038	0.095 ± 0.043	0.106 ± 0.046	0.01 ± 0.006	NA
OP	Triacylglycerol (48:3) levels	X-17357 levels	−0.084 ± 0.038	−0.123 ± 0.038	−0.086 ± 0.036	0.011 ± 6.006	NA
OP	Triacylglycerol (48:3) levels	Cortisone to cortisol ratio	−0.084 ± 0.038	−0.078 ± 0.034	−0.105 ± 0.05	0.008 ± 0.058	NA
OP	Triacylglycerol (51:4) levels	N6-methyllysine levels	−0.09 ± 0.043	0.071 ± 0.034	−0.056 ± 0.021	−0.004 ± 0.003	0.044 (0.075–0.014)
OP	Triacylglycerol (51:4) levels	Spermidine to 5-methylthioadenosine (MTA) ratio	−0.09 ± 0.043	−0.072 ± 0.032	0.1 ± 0.049	−0.007 ± 0.005	0.081 (0.141–0.02)
OP	Triacylglycerol (51:4) levels	Spermidine to choline ratio	−0.09 ± 0.043	−0.094 ± 0.034	0.112 ± 0.054	−0.011 ± 0.007	0.118 (0.194–0.042)

CI = confidence interval, OP = osteoporosis, SE = standard error.

**Figure 4.**
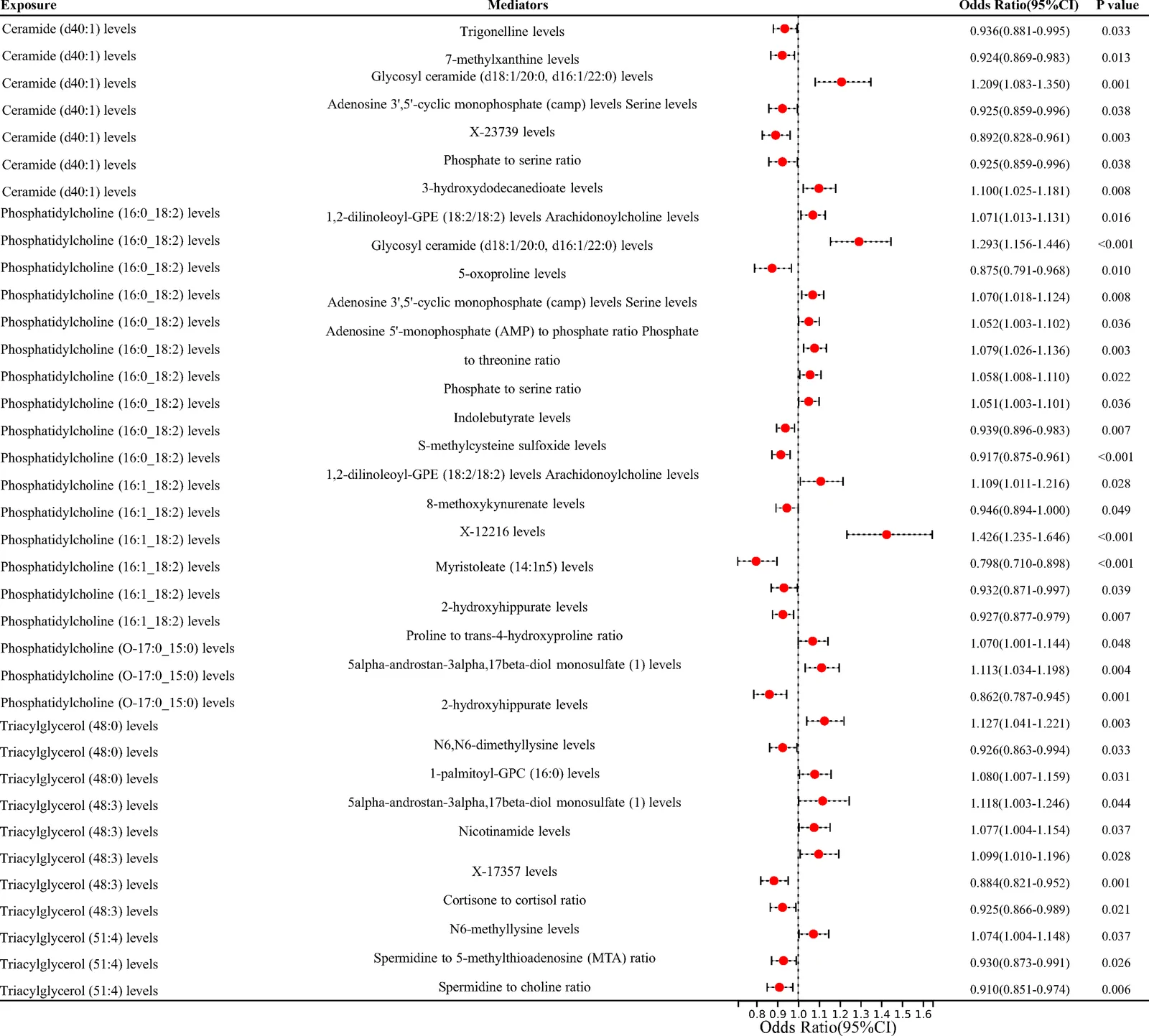
The forest plot of MR analyses the causal effect of plasma lipidomes on plasma metabolites. Data are presented as beta coefficients with 95% confidence intervals (CIs) for the association between each plasma lipidome and candidate mediator metabolites. The inverse variance weighted (IVW) method was used as the primary analysis. Error bars represent 95% CIs. Only metabolites with nominal significance in the mediation analysis are shown. MR = Mendelian randomization.

**Figure 5.**
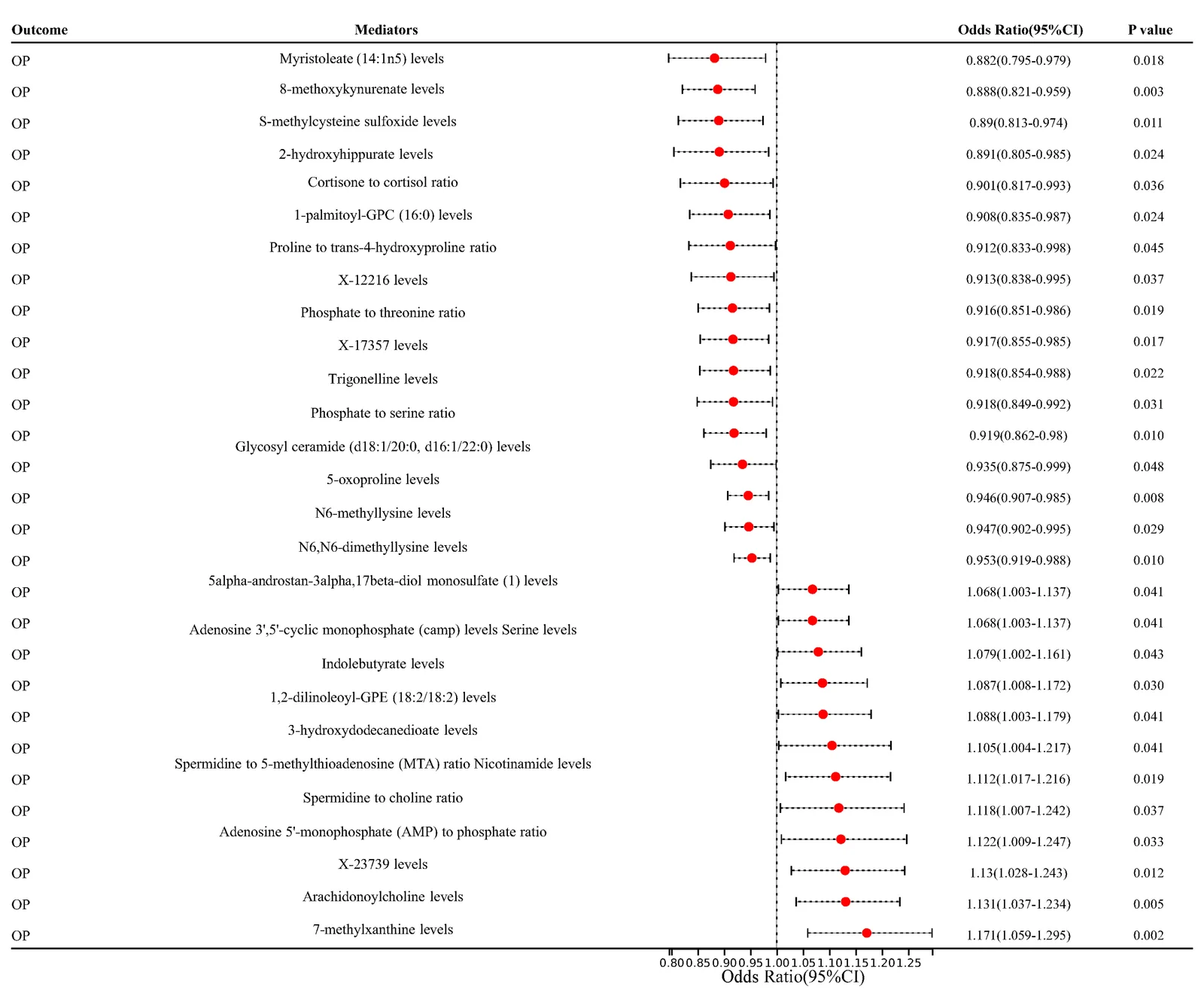
The forest plot of MR analyses the causal effect of plasma metabolites on OP. Data are presented as odds ratios (OR) with 95% confidence intervals (CI) for the association between each candidate mediator metabolite and osteoporosis risk. The inverse variance weighted (IVW) method was used as the primary analysis. Error bars represent 95% CIs. Only metabolites with nominal significance in the mediation analysis are shown. MR = Mendelian randomization, OP = osteoporosis.

## 4. Discussion

In the present large-scale MR study, 7 plasma lipidomes were causally associated with OP. We suggest that ceramide (d40:1) levels, phosphatidylcholine, phosphatidylcholine (16:1_18:2) levels, triacylglycerol (48:3) levels, and triacylglycerol (51:4) levels were negatively associated with OP, whereas phosphatidylcholine (O-17:0_15:0) levels and triacylglycerol (48:0) levels were positively associated with OP. Regarding a possible underlying mechanism, we uncovered 14 plasma metabolites associated with the 7 plasma lipidomes and OP using two-step MR as mediation analyses.

The observed protective effects of certain ceramides and phosphatidylcholines are intriguing. Ceramides are bioactive sphingolipids that participate in cell signaling, including apoptosis and inflammation, which are relevant to osteoblast and osteoclast function.^[19]^ Phosphatidylcholines are major membrane phospholipids that can influence membrane fluidity and signaling cascades.^[20]^ Triacylglycerols, as energy storage molecules, may reflect overall metabolic health, and their association with OP could be mediated by adiposity or insulin resistance.^[21]^ The fact that different lipid species show opposite directions of association underscores the complexity of lipid metabolism in bone pathophysiology.

From metabolomics to genomics, from molecular mechanism to clinical application, these studies have focused on the application of osteoporosis research, revealing the potential effect of plasma metabolites on the risk of osteoporosis, as well as the association between bone turnover markers and metabolites. Wang et al identified potential plasma metabolites in postmenopausal osteoporosis patients using a feature selection approach.^[22]^ Zhao et al discussed the application of metabolomics in osteoporosis research, highlighting the importance of metabolite analysis for understanding disease mechanisms.^[23]^ Di et al explored the relationship between metal mixtures and the risk of osteoporosis by plasma metabolite analysis.^[7]^ Bellissimo et al found an association between serum bone turnover markers and specific metabolites.^[24]^ Weivoda et al identified factors that promote the coupling of bone resorption and bone formation in humans, revealing a link between bone and energy metabolism.^[25]^ Charoenphandhu et al investigated the response of primary bone cells to 1, 25-dihydroxyvitamin D3 in a hyperglycemic environment in a β-globin gene knockout thalassemia mouse model, revealing the link between thalassemia and abnormal bone metabolism.^[26]^ Bellissimo et al found an association between bone turnover markers in serum and specific metabolites through metabolomics analysis, providing clues for the biomarkers of osteoporosis.^[24]^ Weivoda et al identified factors that promote the coupling of bone resorption and bone formation, revealing a link between bone and energy metabolism, and providing a new target for the treatment of osteoporosis.^[25]^ Werdyani et al used plasma metabolomics analysis to identify different endophenotypes of primary osteoarthritis, providing a basis for understanding the heterogeneity of osteoarthritis and individualized treatment.^[26,27]^ Cabrera et al studied the effect of glucocorticoid-induced osteoporosis on plasma metabolome and lipidome in postmenopausal sheep, revealing metabolic changes in osteoporosis.^[28]^ Chen et al constructed a plasma metabolome genomic map and identified metabolites relevant to human disease, providing clues to the molecular mechanism of osteoporosis.^[13]^ Studies by Fan et al and Chen et al reveal potential changes in different activity levels of RA through a multi-omics approach, providing a new perspective for understanding the association of osteoporosis with other inflammatory diseases.^[29,30]^ Wei et al studied the relationship between metabolites related to gut microbes and hand osteoarthritis, suggesting a possible role of gut microbes in osteoporosis.^[31]^ Forrest et al and Behera et al investigated changes in the tryptophan metabolic pathway and the protective effect of hydrosulfide in osteoporosis after drug treatment, respectively.^[32,33]^ Tavel et al evaluated the heterogeneity of osteolytic lesions in patients with multiple myeloma by NMR metabolomics.^[34]^ Porras et al elucidated the mechanism of action of the drug alendronate in the treatment of osteoporosis by inhibiting bone resorption and increasing bone mineral density to reduce the risk of fracture by studying its pharmacokinetic properties.^[35]^ Kunihiro et al proposed a new treatment for osteoporosis from the perspective of inhibiting bone metastatic cancer cells and revealed the potential mechanism of curcumin in inhibiting bone metastatic breast cancer, providing new ideas for the treatment of osteoporosis-related cancers.^[36]^ Ohtomo et al studied the effects of soy isoflavone metabolites on bone mineral density and lipid metabolism in postmenopausal mice, showing the potential of phytoestrogen analogs in the treatment of osteoporosis.^[37]^ Studies by Kassahun et al and Blum et al investigated the metabolism of owl K inhibitors in humans and rats, respectively, and provided important information for drug development in osteoporosis.^[38,39]^ Shandala et al investigated the role of osteocyte apoptosis in cancer chemotherapy-induced osteoporosis, revealing the mechanism of treating-related osteoporosis.^[40]^ The study by Papiha et al found that the vitamin D-binding protein gene was associated with osteoporosis in men, highlighting the importance of genetic variation in osteoporosis.^[41]^ Narayanan et al described the characteristics of avascular necrosis of the femoral head in detail through various methods, providing new insights into the understanding of bone tissue changes associated with osteoporosis.^[42]^ Cabrera et al showed that plasma lipids and polar metabolites were associated with low BMD in Singaporean Chinese postmenopausal women, pointing to the potential application of metabolomics in osteoporosis risk assessment.^[43]^ Studies by Higashi et al and Galvez-Fernandez et al focused on the determination of vitamin D-related compounds and the relationship between REDOX genes, metals, and bone fragility, respectively, further emphasizing the importance of biomarkers in osteoporosis research.^[44,45]^

These research findings suggest that plasma lipidomes may modulate the risk of developing OP through the interaction with other crucial mediators, such as plasma metabolites, either amplifying or decreasing the susceptibility to OP. This mode of action is likely achieved through the regulation of osteoblast and osteoclast activities, thereby further modulating bone formation and bone resorption.

Our investigation encompasses several notable strengths, which are crucial in providing robust evidence on the causal relationship between exposure and the risk of OP, thereby bearing important clinical implications. These strengths include the utilization of a MR design, integration of diverse data sources, bidirectional MR analysis, and implementation of mediation analyses. Together, these methodological approaches enable a comprehensive exploration of causality and facilitate a deeper understanding of the intricate mechanisms underlying OP development and progression. Nevertheless, our study has several limitations. Firstly, the analyses were restricted to individuals of European ancestry, limiting generalizability to other populations. Secondly, despite using MR-Egger and MR-PRESSO to detect and correct for horizontal pleiotropy, we cannot completely rule out residual or undetected pleiotropy, which is an inherent limitation of all MR studies. Thirdly, the two-step MR mediation assumes no unmeasured confounding between the mediator and outcome, which may not hold fully. Then, multiple testing correction was applied only to the primary analyses; the mediation analyses are exploratory and may be subject to false positives. Therefore, the identified mediators should be considered candidate markers rather than confirmed targets.

## 5. Conclusion

In conclusion, our study provides suggestive evidence that specific plasma lipidomes are causally associated with osteoporosis, and that these associations may be partly mediated by circulating metabolites. The modest effect sizes highlight the multifactorial nature of OP and emphasize the need for further biological and clinical investigation. Our findings offer new hypotheses regarding the metabolic pathways linking lipids to bone health and point to potential biomarkers for risk stratification.

## Author contributions

**Data curation:** Hao Lv, Yao Zhou, Zhimu Hu.

**Funding acquisition:** Ting Jiang.

**Software:** Hao Lv, Yan Wang.

**Writing – original draft:** Wenyu Gao, Hao Lv, Qingsong Chu.

**Writing – review & editing:** Wenyu Gao, Jiuxiang Wang, Ting Jiang.






