# Supplementary material for: Role of metabolites in mediating the effect of lipidomes on osteoporosis: A Mendelian randomization study
Source: Medicine (Baltimore). 2026 Nov 6;105(45):e51393. doi: 10.1097/MD.0000000000051393 (PMC13747545; doi:10.1097/MD.0000000000051393)
Supplement: Supplementary file 1 [file medi-105-e51393-s001.docx]

| **Outcome** | **Source** | **GWAS ID/Phenocode** | **Population** | |
| --- | --- | --- | --- | --- |
| Osteoporosis | FinnGen | finngen_R10_M13_OSTEOPOROSIS | European |  |
| plasma lipidomes | PMID:37907536 | GCST90277238-GCST90277416 | European |  |
| plasma metabolites | PMID:36635386 | GCST90199621-GCST90201020 | European |  |
